# Metacognitive therapy versus cognitive–behavioral therapy in adults with generalized anxiety disorder: A 9‐year follow‐up study

**DOI:** 10.1002/brb3.2358

**Published:** 2021-09-14

**Authors:** Stian Solem, Adrian Wells, Leif Edward Ottesen Kennair, Roger Hagen, Hans Nordahl, Odin Hjemdal

**Affiliations:** ^1^ Department of Psychology Norwegian University of Science and Technology Trondheim Norway; ^2^ Faculty of Biology Medicine and Health School of Psychological Sciences Manchester Academic Health Science Centre The University of Manchester Manchester UK; ^3^ Department of Psychology University of Oslo Oslo Norway; ^4^ Research Institute, Modum Bad Vikersund Norway; ^5^ Department of Mental Health Norwegian University of Science and Technology (NTNU) Trondheim Norway

**Keywords:** anxiety disorders, cognitive–behavioral therapy, generalized anxiety disorder, long‐term follow‐up, metacognitive therapy, randomized controlled trial

## Abstract

**Objective:**

Metacognitive therapy (MCT) and cognitive–behavior therapy (CBT) are effective treatments for generalized anxiety disorder. In this study, we followed‐up patients who had previously participated in a randomized controlled trial of MCT compared against CBT.

**Method:**

We collected 9‐year follow‐up data on 39 out of 60 original patients (i.e., 65% response rate).

**Results:**

At 9 years, the recovery rates were 57% for MCT and 38% for CBT (completer analysis). Following MCT, 43% maintained their recovery status and a further 14% achieved recovery. Following CBT, the sustained recovery rate was 13%, while a further 25% achieved recovery. Patients in the MCT condition showed significantly more improvement with respect to symptoms of worry and anxiety. In the CBT group, 23.1% were re‐diagnosed with generalized anxiety disorder (GAD) compared with 9.5% in the MCT group.

**Conclusions:**

This follow‐up study showed a continuation of gains in both treatments at long‐term follow‐up, but with outcomes continuing to favor MCT and strengthening its comparative superiority.

## Graphical abstract

We collected 9‐year follow‐up data on patients treated with cognitive–behavioral therapy (CBT) or metacognitive therapy (MCT). At 9 years, the recovery rates were 57% for MCT and 38% for CBT. In conclusion, this follow‐up study showed a continuation of gains in both treatments at long‐term follow‐up, but with MCT strengthening its comparative outcome superiority.

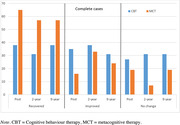

## 1 INTRODUCTION

Generalized anxiety disorder(GAD) is a common, relapsing condition with a poor prognosis if untreated (Portman, 2009). Cognitive behavior therapy (CBT) and metacognitive therapy (MCT) have proven to be effective treatments. Three published randomized trials have compared MCT with various forms of CBT for GAD, and in each case MCT appeared to be more effective (Nordahl et al., 2018; van der Heiden et al., 2012; Wells et al., 2010). Wells et al. (2010) compared MCT with applied relaxation, van der Heiden et al. (2012) compared MCT with CBT based on the intolerance of uncertainty model, and Nordahl et al. (2018) compared MCT with the CBT approach of Borkovec et al. (1993). In each study, MCT was associated with superior outcomes on primary measures and the majority of secondary outcomes. The superiority of MCT was observed over follow‐up periods in these initial studies that ranged from 6 to 24 months. Van der Heiden and Melchior (2014) conducted a 30‐month follow‐up of patients treated in their trial. They showed that both MCT and CBT patients maintained their gains during the interim period from 6‐month to 30‐month follow‐up, with MCT producing a significantly better outcome at 30‐month follow‐up as evidenced by a large between‐group effect (*d *= 1.16) and higher recovery rate (75% vs. 50%).

Little is known concerning the stability of treatment effects in MCT beyond 30 months. One study investigated whether cognitive–behavioral therapy influenced the long‐term (8–14 years) outcome of GAD (Durham et al., 2003), but the study highlighted one well‐known challenge with long‐term follow‐up studies as only 30%–55% attended assessment. However, the results from those who participated suggested that the effect of therapy seemed to be sustained, as 30%−40% of patients recovered.

In the present study, we aimed to evaluate the effects observed at long‐term follow‐up (8–11 years) after completing the trial of Nordahl et al. (2018). In that study, MCT was associated with significantly higher recovery rates (65%) than CBT (38%). Differences favoring MCT were maintained at 2‐year follow‐up. We sought to explore recovery, relapse, changes in symptoms, and diagnostic status of these patients. The main hypothesis was that patients treated with MCT would continue to show more improvement than CBT over the long term. This hypothesis was based on initial responder rates in the original trial (Nordahl et al., 2018), promising long‐term results of MCT for depression (e.g., Hjemdal et al., 2019; Solem et al., 2019), and from a meta‐analysis suggesting that MCT may be more effective than CBT (Normann & Morina, 2018).

## 2 METHOD

### 2.1 Participants and procedure

In the present study, 28 patients were randomized to CBT, and 32 to MCT. At 2‐year follow‐up, there were four patients missing, two in each condition (total *N* = 56, 93.3% attendance rate). At 9‐year follow‐up, there were a total of 39 patients who participated, 17 (60.7%) from the CBT condition and 22 (68.8%) from the MCT condition (total participation rate of 65%). We were unable to reach 15 (25%) patients, five (8.3%) declined to participate, and one was deceased. Of the 39 who participated, 32 completed both questionnaires and interviews, five only completed the questionnaires, and two completed the interviews but did not hand in the questionnaires. The sample had a current mean age of 48.06 (11.70), with no significant age difference between the two conditions (*p* = .52). There were no sex differences (*p* = .36), and 66.7% were women.

It had been 8–11 years since patients completed treatment, with a mean of 9.1 (*SD* = 1.2) years. Interviews took place from January 2018 to June 2019. The follow‐up study was approved by the Regional Committee for Medical and Health Research Ethics (ref no. 2016/1355) and the original trial was registered in Clinicaltrials.gov (identifier: NCT00426426).

Six clinical psychologists, trained in both CBT and MCT delivered the therapy based on published manuals for a maximum of 12 weekly 60‐min sessions. Three therapists delivered CBT first and another three therapists delivered MCT for the first half of the trial before switching conditions. CBT followed the manual by Borkovec and Costello (1993) and consisted of four modules: detecting early cues of anxiety and worry, applied relaxation as a response to these cues, imaginal rehearsal of coping methods with self‐control desensitization, and CBT for catastrophic beliefs and worry. MCT followed the manual by Wells (2009) and consisted of five modules: case formulation and socialization, modifying beliefs about uncontrollability and danger of worry, challenging positive beliefs about the utility and advantages of worry, implementation of alternative coping strategies, and finally relapse prevention.

**FIGURE 1:**
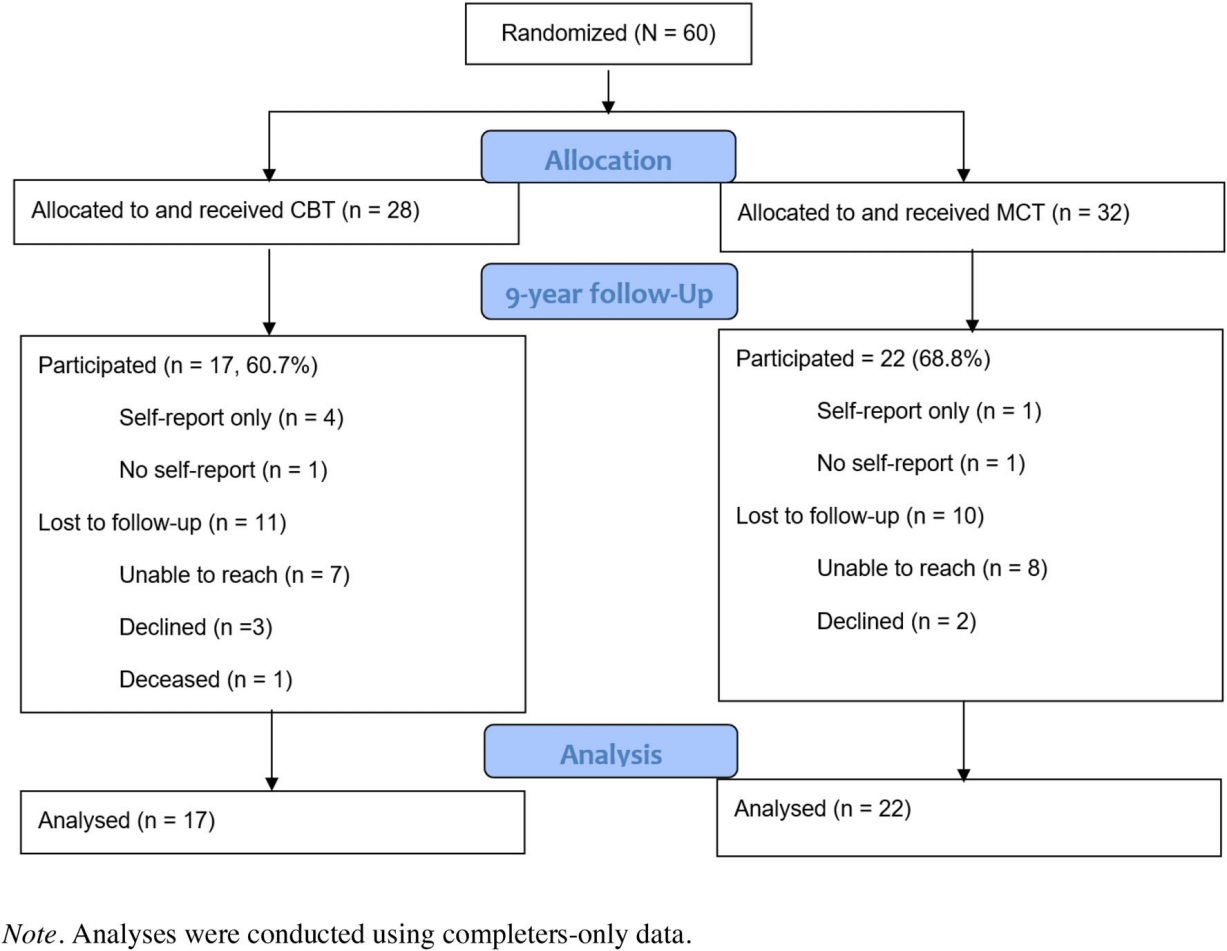
Flow chart describing attrition at follow‐up assessment

Participants were adults diagnosed with GAD who had given written consent to participate. The Anxiety Disorders Interview Schedule for DSM (Di Nardo et al., 1994) was used for diagnostic interviewing. For the follow‐up interviews, the assessment team did not use the entire interview, but selected parts of the interview based on the patients’ diagnoses at pretreatment. Figure 1 summarizes the participant flow from pretreatment to follow‐up.

### 2.2 Measures

The primary outcome measure was the Penn State Worry Questionnaire (PSWQ; Meyer et al., 1990). The PSWQ has 16 items rated on a 1–5 scale, with higher scores indicating higher levels of worry. Scores from 16–39 are considered low, 40–59 moderate, and 60–80 high.

A range of secondary outcomes were used in the initial study including the Beck Anxiety Inventory (BAI), Beck Depression Inventory (BDI), trait‐anxiety, and measures of psychological processes. In the present study, we restricted secondary measures to reduce patient burden and facilitate completion rate. We retained two symptom measures (BAI and BDI).

The BAI (Beck & Steer, 1990) is a 21‐item self‐report inventory assessing anxiety symptoms. Each item is rated on a 0–3 scale. A BAI total score of 0−7 indicates minimal anxiety, 8−15 mild anxiety, 16−25 moderate anxiety, and 26−63 severe anxiety. The BAI was included as a secondary outcome measure.

The BDI (Beck et al., 1961) is a 21‐item self‐report depression inventory. Each item is rated on a 0–3 scale. A BDI total score of 0−9 indicates no depression, 10−18 mild depression, 19−29 moderate depression, and 30−63 severe depression. The BDI was included as a covariate in the repeated measures analyses.

### 2.3 Statistical analyses

Of the 39 who participated, 32 completed both questionnaires and interviews, five only completed the questionnaires, and two completed the interviews but did not hand in the questionnaires. The first statistical tests compared attendees versus nonattendees on scores at pre‐ and posttreatment as well as 2‐year follow‐up. Chi‐square tests and *t*‐tests were used to compare these two groups. Two split‐plot repeated measures ANOVAs were used to explore the effect of treatment and possible time × condition effects on PSWQ and BAI (with BDI as a covariate). Analyses were conducted using complete cases only. We did not impute data as it would not be reliable given the participation rate. Descriptive statistics were used for recovery and relapse rates, and diagnostic status. Criteria used for classifying treatment response were based on the Jacobson and Truax (1991) criteria applied to the PSWQ: clinically significant change/recovery = scoring 47 or less and at least 7‐point improvement on PSWQ; improved = improvement of at least 7 points on PSWQ (reliable change); no change = not achieving reliable change. Other studies have used higher cut‐off values, but 47 was chosen in this study to represent a conservative evaluation of treatment effect, and because it was used in a review on the efficacy of psychological treatments for GAD (Fisher, 2006). This will also allow for easier comparisons across studies.

## 3 RESULTS

### 3.1 Preliminary analyses

Patients who attended the follow‐up assessment were compared with patients who did not participate. We found no significant difference between these two groups on PSWQ and BAI at pretreatment, posttreatment, and 2‐year follow‐up (see Table 1). Furthermore, there was no significant difference between the two groups with respect to posttreatment recovery rates. Also, we found no significant difference between the two groups on age, sex, or number of diagnoses at pre‐ and posttreatment.

**TABLE 1:** Comparisons of participants attending and not attending the assessment at 9‐year follow‐up

	Participants	Nonparticipants	*t*	*p*
PSWQ				
Pretreatment	65.72 (8.27)	67.14 (6.20)	0.691	.492
Posttreatment	48.36 (14.37)	48.52 (15.49)	0.041	.967
2‐Year follow‐up	48.87 (15.49)	49.19 (14.50)	0.078	.938
BAI				
Pretreatment	23.46 (11.59)	22.33 (13.54)	−0.339	.736
Posttreatment	7.38 (7.59)	7.90 (13.47)	0.192	.848
2‐year follow‐up	9.67 (10.10)	10.38 (7.66)	0.283	.778

Abbreviations: BAI, Beck Anxiety Inventory; PSWQ, Penn State Worry Questionnaire.

### 3.2 Changes in symptoms

Two split‐plot repeated measures ANOVAs with Greenhouse–Geisser correction were conducted using 9‐year follow‐up scores on PSWQ and BAI. The results showed a clear effect over time for both measures. Symptoms were significantly reduced from pre‐ to posttreatment, while there were no significant changes in the follow‐up period. There were significant time × condition effects for worry and anxiety symptoms, as there was more improvement in the MCT condition. BDI was a significant covariate for the PSWQ analysis (*p* = .031), but not for BAI (*p* = .204). A summary of the analyses is displayed in Table 2.

**TABLE 2:** Repeated measures ANOVA across four times of assessment for the metacognitive therapy (MCT) and cognitive–behavioral therapy (CBT) group (complete case analyses)

	CBT		MCT		Time	Time × condition		
	*M*	*SD*	*M*	*SD*	*F*	*F*	*p*	*ηp^2^*
PSWQ					9.00***	3.34	.027	.089
Pre	67.31	7.25	64.24	9.22				
Post	54.13	12.19	42.33	13.60				
2‐year	56.44	12.63	41.81	14.82				
9‐year	52.94	13.16	46.95	13.45				
BAI					3.40*	4.22	.011	.110
Pre	20.31	6.99	25.33	14.28				
Post	9.31	8.52	5.00	6.02				
2‐year	12.37	10.75	5.62	6.22				
9‐year	11.44	6.95	8.33	10.48				

Abbreviations: BAI, Beck Anxiety Inventory; PSWQ, Penn State Worry Questionnaire.

*p < .05; ***p < .001.

### 3.3 Recovery and relapse rates

**FIGURE 2:**
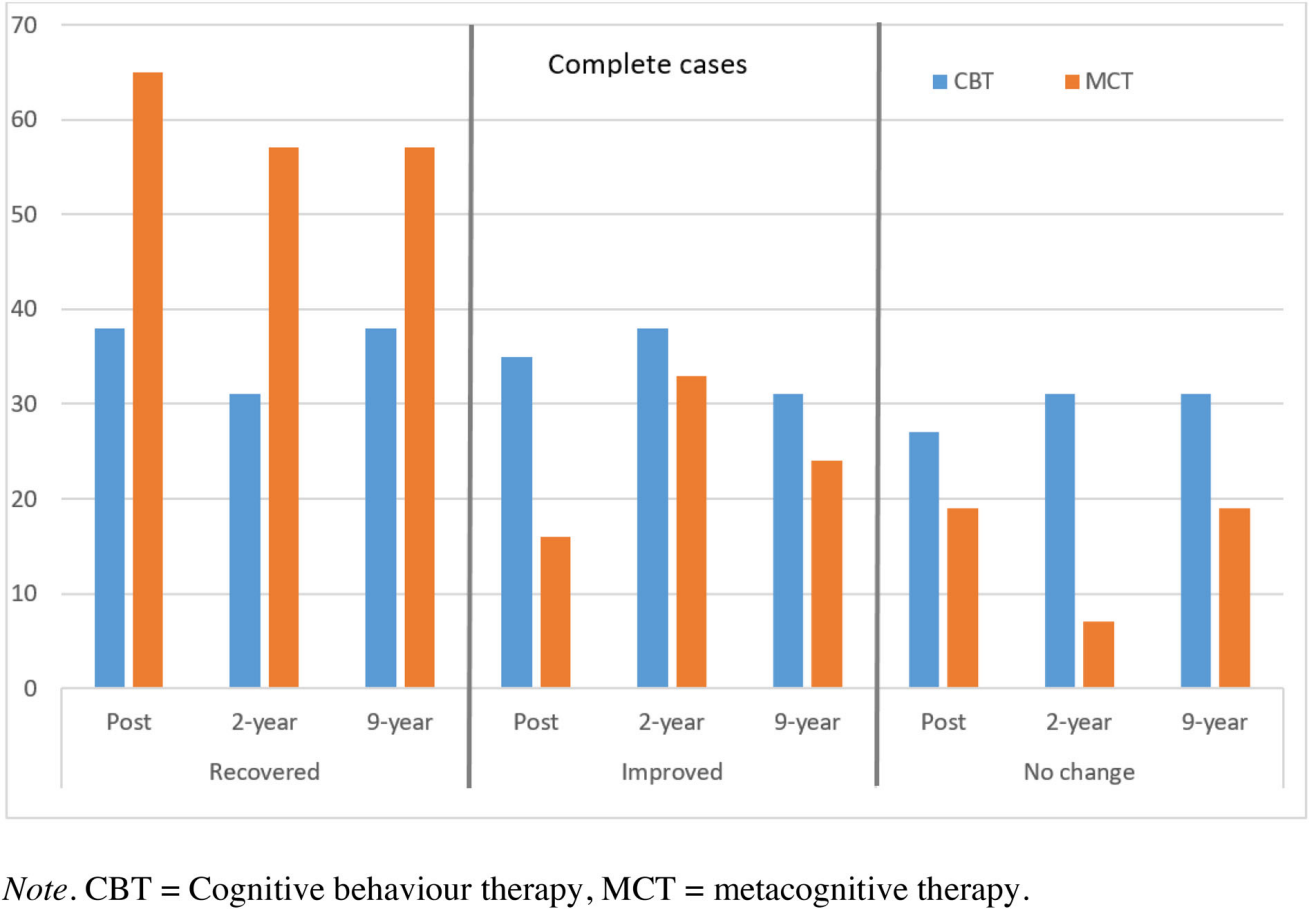
Recovery rates at posttreatment, 2‐year follow‐up, and 9‐year follow‐up

Using complete cases data, the recovery rates were 57.1% for MCT and 37.5% for CBT at 9‐year follow‐up, and the rates of clinical improvement were 23.8% for MCT and 31.3% for CBT. More CBT patients (62.5%) scored above cut‐off (47 points) on PSWQ than MCT patients (42.9%). At pretreatment, all patients scored above 47 on the PSWQ. A summary of comparative recovery rates is displayed in Figure 2.

A summary of recovery rates at posttreatment and follow‐up is displayed in Table 3. Recovery rates were similar at posttreatment and follow‐up. However, while some maintained their recovery status as indicated in Table 3, others achieved recovery, and some relapsed (14% for MCT vs. 19% for CBT relapsed).

**TABLE 3:** Recovery rates at posttreatment and 9‐year follow‐up

	Posttreatment		9‐year follow‐up					
	Status		Status maintained		Status achieved		Overall rate	
Treatment group	*n*	%	*n*	%	*n*	%	*n*	%
MCT (*n* = 21)								
Recovered	13	62	9	43	3	14	12	57
Improved	4	19	2	10	3	14	5	24
No change	4	19	2	10	2	10	4	19
CBT (*n* = 16)								
Recovered	6	38	2	13	4	25	6	38
Improved	5	31	1	6	4	25	5	31
No change	5	31	2	13	3	19	5	31

Abbreviations: CBT, cognitive–behavioral therapy; MCT, metacognitive therapy.

### 3.4 Diagnostic status at follow‐up

Five patients participating in the follow‐up study were not assessed for diagnosis as they only answered the questionnaires. In the CBT group, 23.1% were re‐diagnosed with GAD compared with 9.5% in the MCT group. In the CBT group, 69.2% were not given a diagnosis compared with 81.0% not meeting diagnostic criteria in the MCT group. Two patients were diagnosed with recurrent depression (one moderate and one in remission), and one with social anxiety disorder. In addition, two of the patients had comorbid disorders (dysthymia and fibromyalgia [diagnosed by another institution]).

## 4 DISCUSSION

This study set out to explore the possible long‐term effects of CBT and MCT for patients with GAD. Participation rate was 65% of the original sample. The long‐term recovery rate for CBT was 38%, while for MCT it was higher at 57%. Patients had more improvement in both worry and anxiety in the MCT condition. Following MCT, 43% maintained their recovery status and a further 14% achieved recovery. Following CBT, the sustained recovery rate was 13%, while a further 25% achieved recovery.

The participation rate at follow‐up of 65% is better than other studies, but the figure means that the overall sample is small leading to uncertainty over the reliability of the findings. However, we were unable to find significant differences between participants and nonparticipants on any clinical or demographical variables. This suggests that the follow‐up sample is likely to be representative of the sample that completed treatment.

The advantage of MCT over CBT observed at posttreatment and medium‐term follow‐up was maintained at 9‐year follow‐up. Moreover, this difference appears to have increased when considering the proportions that sustained their recovery status since posttreatment. The difference in outcomes at long‐term follow‐up may reflect different degrees of change in underlying psychological mechanisms. While CBT focuses on developing relaxation skills and challenging the content of worry, MCT has a very different focus. In MCT, the therapist works on challenging beliefs about worry but not the content of worry and helps the patient discover how to regulate worry processes in a way that de‐emphasizes the importance of thoughts. Thus, the better outcome in MCT might conceivably be due to greater change in dysfunctional metacognitions that are a basis for long‐term mental regulation (Wells, 2019).

A study on 30‐month follow‐up for MCT and intolerance of uncertainty therapy (IUT; van der Heiden & Melchior, 2014) reported a recovery rate of 75% for MCT and 50% for IUT. However, the study used different criteria for recovery (cut‐off point ≤ 53; reliable change index ≥ 7). When using the same criteria, the present study has a recovery rate of 67% for MCT and 44% for CBT which is quite comparable, and the consistency supports a superiority of effects observed in MCT. The implication of the MCT versus IUT trial and other studies of MCT, combined with the current results is that MCT should be considered an effective treatment for people with GAD and that treatment effects are probably long‐standing.

The present results are important because they are the first to indicate long‐term follow‐up for MCT against CBT. However, the analysis has major limitations. First, the original sample size combined with the modest follow‐up participation rate means that the follow‐up sample is small and the results may be unreliable. Second, the assessment team did not use the full‐length diagnostic interview (nor SCID‐II interviews), but instead used selected parts of the interview based upon the patients’ diagnoses at pretreatment. Previous research has suggested that GAD could be replaced by somatization disorders (Rubio et al., 2007). Therefore, the current study leaves the question of how many participants developed new disorders unanswered.

In conclusion, the present study showed that both CBT and MCT are associated with long‐term effects and that the superiority of MCT seen at posttreatment may become stronger over long‐term follow‐up. While there appears to be a greater and more stable recovery of MCT patients compared to those who received CBT, this should be confirmed in future studies with larger sample sizes. These results add to the growing body of data in GAD and other disorders showing that MCT and CBT differ in their levels of effectiveness.

## CONFLICT OF INTEREST

Adrian Wells wrote the treatment protocol in MCT and several books on CBT and MCT, and received royalties from these. All other authors declare no competing interests.

### PEER REVIEW

The peer review history for this article is available at https://publons.com/publon/10.1002/brb3.2358

## Data Availability

The data that support the findings of this study are available from the corresponding author upon reasonable request.
